# Credible Mendelian Randomization Studies in the Presence of Selection Bias Using Control Exposures

**DOI:** 10.3389/fgene.2021.729326

**Published:** 2021-11-24

**Authors:** Zhao Yang, C. Mary Schooling, Man Ki Kwok

**Affiliations:** ^1^ School of Public Health, Li Ka Shing Faculty of Medicine, The University of Hong Kong, Hong Kong, China; ^2^ Graduate School of Public Health and Health Policy, City University of New York, New York, NY, United States

**Keywords:** causal estimates, control exposures, Mendelian randomization, reproducible, selection bias

## Abstract

Selection bias is increasingly acknowledged as a limitation of Mendelian randomization (MR). However, few methods exist to assess this issue. We focus on two plausible causal structures relevant to MR studies and illustrate the data-generating process underlying selection bias *via* simulation studies. We conceptualize the use of control exposures to validate MR estimates derived from selected samples by detecting potential selection bias and reproducing the exposure–outcome association of primary interest based on subject matter knowledge. We discuss the criteria for choosing the control exposures. We apply the proposal in an MR study investigating the potential effect of higher transferrin with stroke (including ischemic and cardioembolic stroke) using transferrin saturation and iron status as control exposures. Theoretically, selection bias affects associations of genetic instruments with the outcome in selected samples, violating the exclusion-restriction assumption and distorting MR estimates. Our applied example showing inconsistent effects of genetically predicted higher transferrin and higher transferrin saturation on stroke suggests the potential selection bias. Furthermore, the expected associations of genetically predicted higher iron status on stroke and longevity indicate no systematic selection bias. The routine use of control exposures in MR studies provides a valuable tool to validate estimated causal effects. Like the applied example, an antagonist, decoy, or exposure with similar biological activity as the exposure of primary interest, which has the same potential selection bias sources as the exposure–outcome association, is suggested as the control exposure. An additional or a validated control exposure with a well-established association with the outcome is also recommended to explore possible systematic selection bias.

## Highlights

### What is Already Known on this Subject?

• Mendelian randomization (MR) provides unconfounded estimates, but is particularly vulnerable to selection bias because of the small magnitude of genetic estimates.
• Negative controls provide helpful tools to detect residual confounding, selection, and measurement bias in conventional epidemiological studies but often lack specificity in the type of bias they detect.

### What this Adds to What is Known?

• Given genetics are a lifelong exposure, a key source of selection bias in MR studies is missing people from the same underlying birth cohorts as the original population who die before recruitment, which may violate the exclusion-restriction assumption and distort the MR estimates.
• The use of control exposures that have the same potential selection bias sources as the exposure–outcome association of interest can detect potential selection bias and validate MR estimates.
• The estimated exposure–outcome association is more credible if this result is robust to potential selection bias and reproducible by using the relevant control exposures based on subject matter knowledge.

### What is the Implication, What Should Change Now?

• Systematic selection bias may occur particularly when the genetic variants affect survival and the outcome of interest or a competing risk of that outcome affects survival; interpretation of MR estimates should be cautious.
• The routine use of control exposures could add more credibility to MR estimates.

## Introduction

**FIGURE 1:**
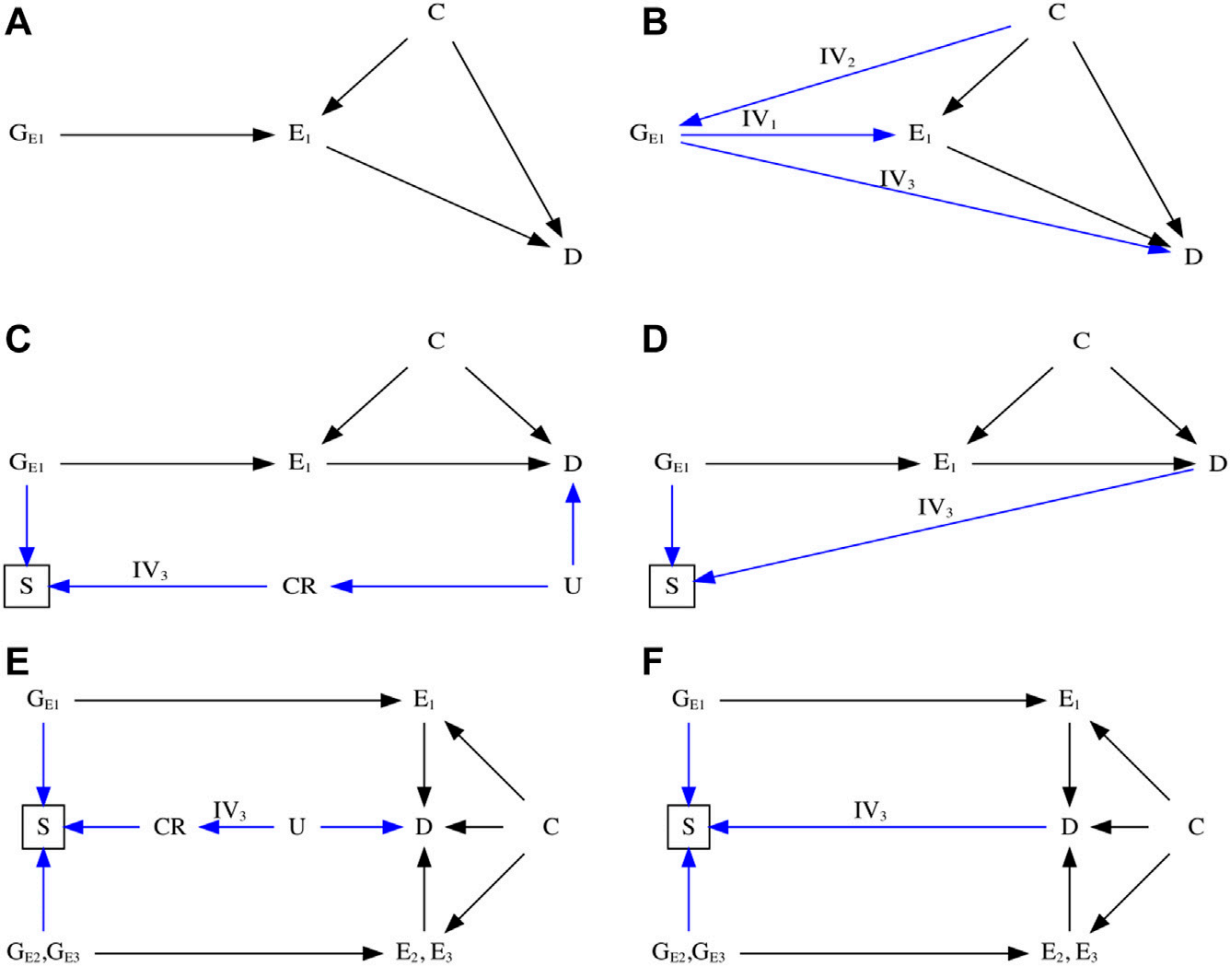
Directed acyclic graph (DAG) illustrating Mendelian randomization (MR). **(A)** DAG illustrating an ideal scenario of an MR study. **(B)** DAG illustrating the three instrumental assumptions (Davey Smith and Ebrahim, 2003)—IV1: Relevance (Burgess et al., 2015); IV2: Independence (Burgess et al., 2012); IV3: Exclusion restriction. **(C)** DAG illustrating potential biased pathway with selection bias in the presence of competing risks that share substantial etiological factors with the outcome. **(D)** DAG illustrating potential biased pathway with selection bias in the unrepresentative selected samples. **(E)** DAG illustrating an MR study using control exposures to detect potential selection bias in the presence of competing risks. **(F)** DAG illustrating an MR study using control exposures to detect potential selection bias in the unrepresentative selected samples. E_1_: the primary exposure of interest; E_2_ and E_3_: the control exposures; C: the confounder that associates with both the exposure and outcome; D: the outcome; CR: the competing risks; U: the unmeasured and shared confounders of the competing risks and the outcome; G_E1_, G_E2_, and G_E3_: genetic variants that are strongly associated with the exposure of primary interest and the control exposures.

Mendelian randomization (MR) uses genetic variants as a natural experiment in observational studies to investigate potential causal effects of modifiable risk factors on health outcomes (Davey Smith and Ebrahim, 2003). MR is often conducted in two homogeneous study populations, i.e., two-sample MR (Burgess et al., 2015). MR is thought to be robust to the confounding that often occurs in conventional observational studies due to the random allocation of genetic endowment at conception being used as a proxy for the exposure (Burgess et al., 2012; Davies et al., 2018). Currently, MR is a popular approach for assessing causality (Sekula et al., 2016). However, MR estimate rests on stringent assumptions, as illustrated using directed acyclic graphs (DAGs) in Figures 1A,B (Davey Smith and Ebrahim, 2003; Lawlor et al., 2008).

• IV1 (the relevance assumption): the genetic variant is robustly associated with the exposure of interest;
• IV2 (the independence assumption): the genetic variant is not associated with confounders that bias the exposure–outcome association;
• IV3 (the exclusion-restriction assumption): the genetic variant affects the health outcome only *via* its effect on the exposure.

Notably, aside from IV1 that can be empirically verified using the F-statistic (Staiger and Stock, 1997; Bowden et al., 2016a), IV2 and IV3 are typically harder to justify. Hence, violations of these assumptions can occur, leading to misleading conclusions. Of these, selection bias is increasingly acknowledged as distorting MR estimates in the selected populations investigated (Nitsch et al., 2006; VanderWeele et al., 2014; Canan et al., 2017; Vansteelandt et al., 2018a; Vansteelandt et al., 2018b; Munafò et al., 2018; Munafò and Smith, 2018; Gkatzionis and Burgess, 2019; Smit et al., 2019; Schooling et al., 2020) and has largely focused on bias arising from selection on exposure (Vansteelandt et al., 2018a; Vansteelandt et al., 2018b; Munafò et al., 2018; Gkatzionis and Burgess, 2019; Smit et al., 2019).

Genetic studies are usually carefully designed to avoid selecting sample on genetic make-up and phenotypes. Generally, selection bias occurs in an MR study when the sample in the original genome-wide association study (GWAS) are selected conditional on survival until study recruitment on genotype of interest in the presence of prior death from the outcome or competing risks of the outcome (Figure 1C), especially in the original outcome GWAS (Schooling et al., 2020). The problem is the time lag between genetic randomization at conception and recruitment of participants into the GWAS. Participants diagnosed with or dead from the outcome or a competing risk of the outcome are not recruited into the outcome GWAS, which attenuates or reverses MR estimates for harmful exposures, because people who have already died of their harmful genetic endowment and people who have died of the outcome or a competing risk of the outcome are missing. As such, selection bias may create a spurious genetic variant–outcome association by opening the backdoor path from genetic instruments to the outcome of interest, violating the IV3 assumption.

**FIGURE 2:**
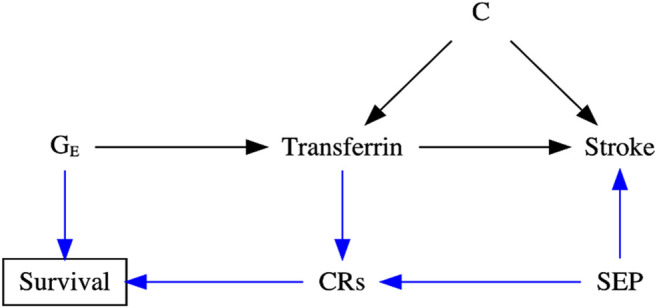
Directed acyclic graph (DAG) illustrating the possible data-generating process underlying selection bias in the transferrin–stroke association due to missing people in the presence of competing risks (CRs, e.g., coronary artery disease) caused by the shared confounder [e.g., socioeconomic position (SEP)] of stroke and CRs in two-sample Mendelian randomization settings. C: the unmeasured confounder of the transferrin–stroke association.

For example, previous observational studies showed that higher transferrin binds to circulating iron and influences iron status, which may further cause iron-deficiency anemia and increase the risk of stroke (Chang et al., 2013; Marniemi et al., 2005; Gillum et al., 1996). However, a recent MR study reported that lower iron status also appeared to protect against stroke (van der et al., 2005; Gill et al., 2018), especially cardioembolic stroke (Gill et al., 2018). An increasingly acknowledged explanation is selection bias, possibly due to the presence of competing risks [e.g., coronary artery disease (Gill et al., 2017), hypercholesterolemia (Gill et al., 2019), chronic kidney disease (Fishbane et al., 2009), skin infections (Gill et al., 2019), liver disorders (e.g., hepatitis C) (Shan et al., 2005), and rheumatoid arthritis (Yuan and Larsson, 2020)] caused by the shared confounders (e.g., socioeconomic position, lifestyle, and health status), affecting survival of the underlying population (Camaschella, 2015; McLean et al., 2009), as shown in Figure 2. For instance, people with competing risks, such as coronary artery disease, tend to die earlier than those with stroke in Western settings (Kesteloot and Decramer, 2008; Menotti et al., 2019; Diseases and Injuries, 2020). As such, people vulnerable to these competing risks with higher iron status may die before study recruitment, leaving more “healthier” participants in the study and inducing biased estimates.

Several statistical methods have been proposed to detect and eliminate selection bias in MR studies, most of which focus on bias arising from selection on exposure (Bareinboim and Pearl, 2012; Arnold and Ercumen, 2016; Hemani et al., 2017; Tchetgen Tchetgen and Wirth, 2017; Vansteelandt et al., 2018a; Brumpton et al., 2020; Zhao et al., 2020; Sanderson et al., 2021; Wang and Han, 2021), which is generally thought to have limited effects. However, selection on genetic endowment and outcome or competing risk of the outcome is more pervasive (Schooling et al., 2020) and can have larger effects. One approach that has not been considered is the use of a “negative control,” which has been widely used in laboratory science for decades to help detect problems with the experimental method (Arnold and Ercumen, 2016). In epidemiological studies, a formal approach has been described in detail and suggested as a means of detecting residual confounding, selection bias, and measurement bias (Lipsitch et al., 2010; Arnold et al., 2016). Recently, negative control outcomes, defined as sharing identical confounders with the exposure–outcome association but not associated with the exposure, have been proposed to detect potential population stratification in MR studies (Sanderson et al., 2021). Other approaches include summary data-based MR [SMR, e.g., MR robust adjusted profile score (MR-RAPS)] (Zhao et al., 2020; Wang and Han, 2021), two-sample MR Steiger method (Hemani et al., 2017), and three-sample MR (Zhao et al., 2019), in which the selection procedure of genetic instrument (e.g., winner’s curse) is considered a form of selection bias (Wang and Han, 2021). However, such a situation is different from the scenario where the original outcome GWAS is missing people from the same underlying population (birth cohorts) as those included, some of whom have already died from the instrument and some of whom have already died from the outcome or a competing risk of the outcome, as shown in Figures 1C,D.

In this study, as an extension of negative control outcomes, we advance the use of control exposures to validate MR estimates that might be susceptible to such selection bias. We focus on plausible causal structures relevant to MR studies and illustrate how to validate MR estimates using control exposures through a real example investigating the potential association of transferrin with stroke (including ischemic and cardioembolic stroke). This association is thought to be particularly vulnerable to selection bias, especially among older populations, because transferrin affects survival and stroke is open to competing risk from IHD (Schooling et al., 2020; Yang et al., 2021). We further discuss the criteria for choosing the control exposures and the limitations of this approach.

## Methods

Figures 1C,D show DAGs for MR with selection bias caused by sample selection. In the presence of competing risks (Figure 1C), the selected samples may have a lower risk of developing the phenotype [e.g., the outcome (D)] because the GWAS is missing people with genetic vulnerability to earlier death and people who have died from a disease that shares causes (e.g., U) with the phenotype. As such, the backdoor pathway directly linking G_E1_ to D will be reopened in the selected samples if the instruments affect survival, i.e., have allele frequencies that differ from the underlying population (e.g., birth cohort). This situation violates the IV3 assumption and distorts MR estimates, which can attenuate or reverse the true association or create a spurious association. The small effect sizes of genetic associations (Park et al., 2011; Global Burden of Disease, 2020) make them particularly vulnerable to perturbation by such bias (Schooling et al., 2020). In the absence of competing risks (Figure 1D), the phenotype (e.g., D) risk and instruments’ frequencies may vary because of selecting on genetic instruments and outcome, which generates unrecoverable selection bias.

To clearly illustrate the data-generating process underlying selection bias due to missing people from the original birth cohorts who formed the underlying population through death before study recruitment, we conducted extensive simulation studies. Details are presented in the Supplementary Material. Briefly, we induced selection bias by selecting study participants as survivors to study recruitment. We assumed that the survival of the underlying population was influenced by the genetic instruments G_E1_, exposure E_1_, outcome D, confounder C of the exposure–outcome association, or the unmeasured confounder U mediated by competing risks CR. We used the relative hazard (i.e., hazard ratio) per-unit change in either G_E1_, E_1_, C, D, or U to quantify their effects on the survival, as shown in Supplementary Figure S1. As such, the impact of selection bias induced by the survival status of the underlying population until study recruitment was governed by hazard ratio of per-unit change in either G_E1_, E_1_, C, D, or U. Then, we induced selection bias in two-sample MR by having instruments determining survival to recruitment and outcome of interest affecting survival to recruitment. Details of the simulation study are in the Supplementary Material, along with the corresponding R scripts.

**FIGURE 3:**
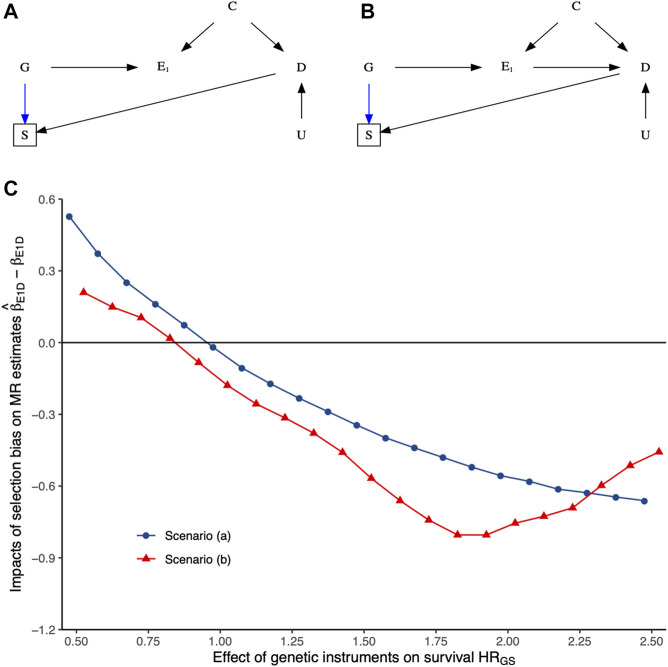
The impacts of selection bias (i.e., ${\hat{\beta }}_{E1D}-\beta_{E1D}$ ) on two-sample Mendelian randomization (MR) estimates of the exposure E_1_–outcome D association using the inverse-variance weighted method in terms of various relative hazards (HRs) of per-unit change in genetic instruments G (i.e., HR_GS_) with a fixed effect of D (i.e., HR_DS_) on survival of underlying population based on simulation studies, with more details presented in Supplementary Material S1. The upper panels **(A, B)** show scenarios that may happen in practice. The lower panel **(C)** shows the impacts of selection bias on MR estimates under each scenario. R codes for reproducing these results can be found in Supplementary Material S2.

Figure 3 and Supplementary Figure S1 show the impact of selection bias arising from selecting samples conditioning on genetic instruments G and outcome D, with no effects of either exposure E_1_ or the shared confounder U mediated by competing risks on survival of the underlying population (i.e., birth cohort) based on simulation studies. More details have been presented in Supplementary Material S1. As expected, selecting samples conditioning on genetic instruments G and outcome D of interest induces selection bias, with its impacts varying depending on the relative hazard of G and D on survival of the underlying population. Given summary statistics obtained from the original exposure and outcome GWASs, it seems not easy to recover the true causal estimate from the observed MR estimates in two-sample MR settings due to the essence of missing people before the recruitment of the original GWASs.

### Validating MR Estimates by Detecting Selection Bias and Reproducing Associations of Interest Using Control Exposures

To explore selection bias, we reproduce a condition that does not involve the hypothesized causal mechanism but involves the same potential selection bias sources in the original MR study. We introduce an antagonist or decoy of E_1_ as the control exposure E_2_, mimicking a natural experiment, because E_2_ acts as an endogenous intervention of E_1_. Moreover, E_2_ effects on survival would be nearly identical to E_1_, as depicted in Figures 1E,F, but has an opposite impact on D from E_1_. If such an E_2_ exists, then any consistent effects of E_1_ and E_2_ on D would be mainly due to selection bias rather than study design. That is, the consistent effects of E_1_ and E_2_ on D could indicate potential selection bias. Otherwise, the estimated causal effects derived from the selected samples are robust to selection bias. Moreover, an intuitive interpretation herein is that the E_1_–D association is credible and reproducible by using a relevant control exposure E_2_ because of the known relationship between E_1_ and E_2_.

We can extend the selection of E_2_ by using exposure with similar biological activity as E_1_ because they are also likely to share the same potential selection bias sources and have similar or even the same effects on D. This idea is widely applied in developing pharmaceutical products [Food and Drug A (2014). Bioa, 2014; Committee for Medicinal P, 2010]. If such an E_2_ exists, then any inconsistent effects of E_1_ and E_2_ on D would be mainly caused by potential selection bias. Conversely, consistent results of E_1_ and E_2_ on D would validate the estimated effects. In other words, these estimated effects derived from the selected samples are less likely to be affected by selection bias. Even if selection bias exists, its impact would be limited. It would not extend to reverse the causal direction or distort the estimated effect far away from the truth. Notably, the use of such kinds of control exposures does not require a null or well-established association between the control exposure E_2_ and D.

### Issue of Systematic Selection Bias

However, this method might still fail to detect selection bias if systematic selection bias exists, especially when E_1_ and E_2_ are selected from the same GWAS. In such a case, it might distort both the E_1_–D and E_2_–D associations similarly, such as reversing the estimated E_1_–D and E_2_–D associations simultaneously. To handle this situation, we introduced an additional negative (or positive) control E_3_ with the same potential selection bias sources concerning the E_1_–D association or identified a validated control exposure (E_2_) that had a clear association with D to triangulate the estimated effects. As such, any associations of E_3_/E_2_ with D would indicate potentially systematic selection bias. Otherwise, the estimated effects derived from the selected samples are likely to be robust to selection bias and reproducible.

### Choosing Control Exposures

Control exposures could be used to detect potential selection bias and validate MR estimates. To this end, it might be necessary to specify the criteria for choosing the control exposures E_2_ and/or E_3_ as follows.

1) The control exposure E_2_ should have the same potential selection bias sources (e.g., affecting survival in the underlying population) as E_1_ on D. For example, using antagonist, decoy, or an exposure with similar biological activity as E_2_, such a criterion is approximately satisfied;
2) To explore potentially systematic selection bias, an additional control exposure (E_3_) with the same potential selection bias sources as E_1_ on D or a validated control exposure E_2_ should have a well-established association with D.

We recommend choosing E_1_, E_2_, and/or E_3_ from different GWASs to minimize potentially systematic selection bias. If such E_2_ and E_3_ exist, then the estimated effects of E_1_, E_2_, and E_3_ on D can be used to detect potential selection bias and triangulate the causal estimates. The estimated E_1_–D association would be more credible because it is robust to potential selection bias and can be reproducible using a relevant control exposure E_2_ based on subject matter knowledge.

### An Applied Example

To illustrate, we investigated the association of higher transferrin (i.e., E_1_) with stroke (including ischemic and cardioembolic stroke), with transferrin saturation as a control exposure E_2_ and iron status as a positive control exposure E_3_. We selected transferrin saturation as the control exposure E_2_ because it measures circulating iron and reflects the proportion of transferrin occupied by iron (Wish, 2006). Biologically, transferrin saturation is inversely associated with transferrin but positively associated with iron status. Furthermore, iron deficiency, reflected by lower transferrin saturation and higher transferrin, causes anemia and reduces lifespan directly or *via* competing risks [e.g., stroke (23), Figure 2] (McLean et al., 2009; Camaschella, 2015). Consequently, the associations of transferrin saturation and iron status with stroke are open to similar potential selection bias as the transferrin-stroke association. Hence, transferrin saturation and iron status are control exposures here. As such, any consistent transferrin–stroke and transferrin saturation–stroke associations (especially in the same causal direction) indicate potential selection bias. In addition, any null iron status–stroke association suggests the presence of systematic selection bias due to its clear associations with stroke and longevity (Gill et al., 2018; Daghlas and Gill, 2021); particularly, the iron status-longevity association is less likely to subject to selection bias (Andersen et al., 2012).

**FIGURE 4:**
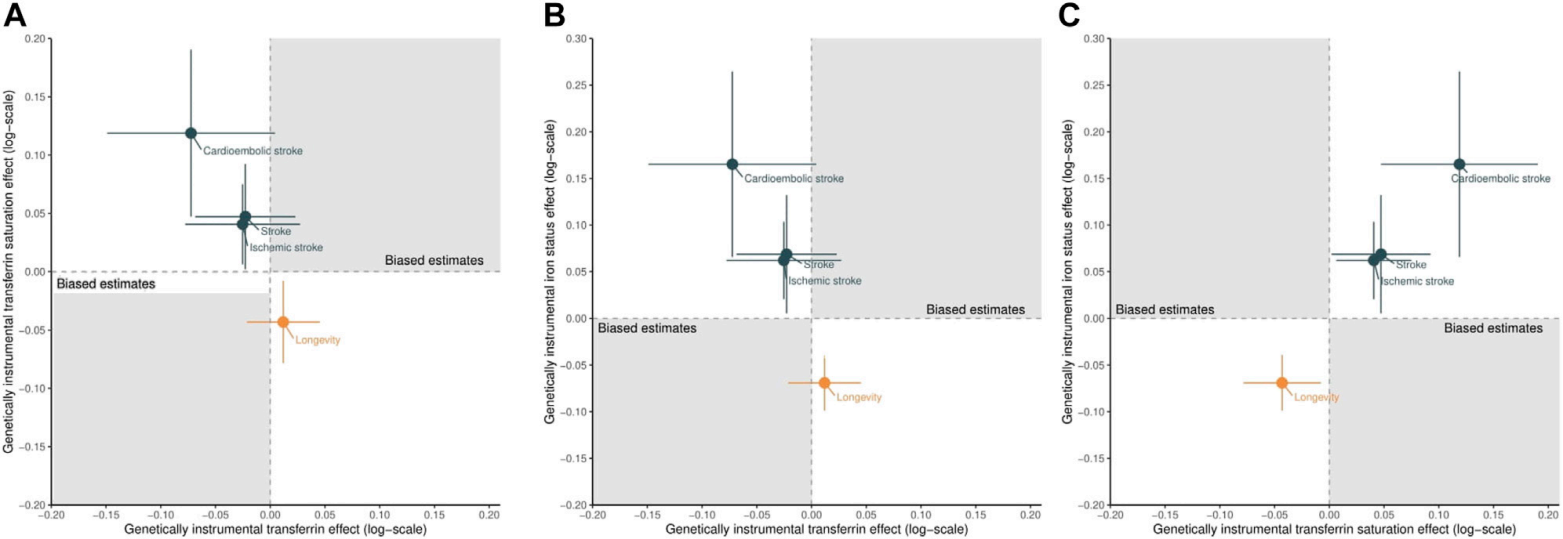
Scatter plots of the estimated effects of genetically predicted higher transferrin versus higher transferrin saturation **(A)**, higher transferrin versus higher iron status **(B)**, and higher transferrin saturation versus higher iron status **(C)** on stroke (including ischemic and cardioembolic stroke) and longevity. Points located in the gray area indicate the presence of selection bias.

We selected independent (r^2^ < 0.01) genetic instruments mimicking effects of transferrin (MR-base id: ieu-a-1052), transferrin saturation (MR-base id: ieu-a-1051), and iron status (MR-base id: ieu-a-1049) from the MR-base at a genome-wide significance $p<5\times 10^{-8}$ (Benyamin et al., 2014). We approximated the F statistics (i.e., the square of instrument’s association on exposure divided by the square of its SE) to assess the instrument strength, where higher F statistics indicate a low risk of weak instrument bias (Bowden et al., 2016a). We excluded the instruments with F statistics less than 10 to alleviate potential weak instrument bias (Bowden et al., 2016a). We checked the shared instruments for transferrin, transferrin saturation, and iron status to explore the possibility of pleiotropic effects, but still used them in this example as they have been used similarly in a previous MR study (Daghlas and Gill, 2021). We further assessed associations of higher transferrin saturation and iron status with longevity, proxied by the heritable trait of parental lifespan from United Kingdom Biobank and LifeGen consortium (Timmers et al., 2019). Genetically predicted higher transferrin saturation and higher iron status were inversely associated with longevity, as shown in Figure 4, suggesting the similar or even the same selection bias sources as the transferrin–outcome association because it also appeared to affect longevity.

We applied the identified instruments to publicly available GWAS of European descent of stroke (40,585 cases and 406,111 controls), ischemic stroke (34,217 cases and 406,111 controls), and cardioembolic stroke (7,193 cases and 406,111 controls) (Timmers et al., 2019). Supplementary Table S1 presents a detailed summary of the included studies. We extracted summary statistics for stroke (MR-base id: ebi-a-GCST005838), ischemic stroke (MR-base id: ebi-a-GCST005834), and cardioembolic stroke (MR-base id: ebi-a-GCST006910) from MR-base (Hemani et al., 2018). Supplementary Table S2 lists genetic associations of the included instruments associated with stroke.

We assessed the associations of genetically predicted transferrin, transferrin saturation, and iron status with stroke using the Wald ratio (i.e., the ratio of the genetic outcome effect estimate and the corresponding genetic exposure effect estimate) or the inverse-variance weighted average of the Wald ratio estimates with random effects (Burgess et al., 2013). We assumed that all these associations were linear and homogeneous (Lawlor et al., 2008). We reported Cochran’s Q-statistic to detect potential heterogeneity. We conducted sensitivity analyses using the weighted median (Bowden et al., 2016b), MR-Egger (Bowden et al., 2015), and MR-RAPS(40) to address the potential unknown pleiotropy statistically. We also reported the MR-Egger intercept and its SE with *p*-value as an indicator of potential pleiotropy. Two-sided *p*-values at the Bonferroni-corrected threshold of 0.05/3 (for three exposures) = 0.017 were considered statistically significant. *P-*values between 0.017 and 0.05 were reported as nominal. Data involving these exemplars were publicly available, so it does not require ethical approval.

## Results

Up to 11 genetic instruments were used for transferrin (mean concentration 2.1 g/L and SD 0.43 g/L), 7 instruments for transferrin saturation (mean percentage 29.9% and SD 11.0%), and 5 instruments for iron status (mean concentration 18.4 μmol/L and SD 5.6 μmol/L). The F-statistics of instruments for transferrin ranged from 32.4 to 1,296.1, for transferrin saturation ranged from 35.6 to 808.5, and for iron status was 37.8 to 346.7, suggesting weak instrument bias to be less likely.

Figure 4 shows the scatter plot of the estimated effects of genetically predicted higher transferrin versus higher transferrin saturation (A), higher transferrin versus higher iron status (B), and higher transferrin saturation versus higher iron status (C) on stroke (including ischemic and cardioembolic stroke) and longevity, with full details presented in Supplementary Table S3. Genetically predicted higher transferrin was associated with a lower risk of stroke (Figures 4A,B), although these protective effects did not reach nominal significance (*p* < 0.05). Conversely, genetically predicted higher transferrin saturation was nominally associated with higher risk of stroke (Figures 4A,C). Such results suggest that the observed transferrin–stroke association is open to selection bias, possibly due to the missing people from the original GWAS of stroke because they died before recruitment from the genetic predictors of iron, an iron-related condition, stroke, or a competing risk of stroke, which attenuated the true association (Figure 2).

In addition, as expected (Gill et al., 2018; Daghlas and Gill, 2021), genetically predicted higher iron status was associated with increased stroke and reduced longevity, as shown in Figures 4B,C and Supplementary Table S3. Finally, the consistent effects of higher transferrin saturation and higher iron status on stroke and longevity further triangulated our conclusions. Even if selection bias exists, its impact on the transferrin saturation–stroke and iron status–stroke associations would be limited or at least could not reverse the observed associations or biased them to the null. These results support the advantages of using control exposures.

## Discussion

This paper advances the use of control exposures based on subject matter knowledge in MR studies to triangulate the estimated causal effects vulnerable to selection bias. The potential mechanisms underlying selection bias in MR lies in the re-opened backdoor pathway from genetic instruments to the outcome of interest in the selected samples. It violates the IV3 assumption and distorts the MR estimates. The applied example demonstrates that MR is vulnerable to selection bias because of missing data from sample selection (Figures 1, 3), which is unlikely to be missing at random, so requires modeling of the missing data process to recover the estimates (Mohan and Pearl, 2021). Our proposal provides a valuable approach to assessing credible MR estimates in the presence of selection bias from selection of survivors*.*

Furthermore, the control exposures introduced in the proposal inherit properties similar to those of negative or positive control exposures used in the conventional observational studies but provide a more intuitive and clinically meaningful interpretation of the estimated effects (Lipsitch et al., 2010; Shi et al., 2020; Sanderson et al., 2021). Choosing antagonists, decoys, or exposures with similar biological activity as the control exposures based on subject matter knowledge may facilitate its application in MR studies. Systematic selection bias distorting both the exposure–outcome and control exposure–outcome associations, in a similar or even the same way, may exist, resulting in inconclusive or misleading conclusions. However, an additional or a validated control exposure with a clear association with the outcome provides another tool to triangulate the estimated effects. Notably, it is possible to use a single control exposure in the proposal solely to validate the MR estimates, especially when E_1_, E_2_, and E_3_ are selected from different GWASs.

Despite the strengths of the proposal in validating MR estimates, limitations exist. First, the proposal only detects potential selection bias but fails to address it. The impact of selection bias on summary statistics obtained from the original GWAS might vary due to the small fraction of heritability explained by genetic variants and the small effect size of the genetic associations (Greenland, 2003; Freedman et al., 2004; Park et al., 2011; Schooling, 2019). Thus, the proposal might fail to detect its small effect on MR estimates. Nonetheless, routinely applying control exposures still adds more credibility to MR estimates. Second, the proposal inherits properties of the conventional MR; limitations such as the stringent instrumental assumptions remain (Davey Smith and Ebrahim, 2003; Smith and Ebrahim, 2004; Lawlor et al., 2008). However, recent advances in MR provide more tools to alleviate or even eliminate these limitations (Ye et al., 2019; Zhao et al., 2020; Liu et al., 2021). Third, choosing control exposures that have the same potential selection bias sources as the exposure–outcome association of interest or a clear association with the outcome might be difficult in practice, further limiting its application.

## Conclusion

Routinely using control exposures in MR studies provides a helpful tool to validate estimated causal effects that are vulnerable to potential selection bias in the selected samples.

## Data Availability

The original contributions presented in the study are included in the article/Supplementary Material; further inquiries can be directed to the corresponding author.

## Abbreviations

CR, competing risks; DAG, directed acyclic graph; GWAS, genome-wide association study; IV, instrumental variable; MR, Mendelian randomization.
