# Supplementary material for: Credible Mendelian Randomization Studies in the Presence of Selection Bias Using Control Exposures
Source: Front Genet. 2021 Nov 24;12:729326. doi: 10.3389/fgene.2021.729326 (PMC8652250; doi:10.3389/fgene.2021.729326)
Supplement: Supplementary file 1 [file DataSheet2.PDF]

```
rm(list=ls())
library(data.table)
library(ivmodel)
library(tcensReg)
## 2SLS
# Y: the outcome
# D: the exposure
# Z: the instrument
# X: the covariates
ivRes <- function(out,exp,ins,cov) {
  ivModel <- ivmodel(Y=out,D=exp,Z=ins,X=cov)
  a <- coef(ivModel)
  c <- confint(ivModel)
  data.frame(method="TSLS",
           betaXY=a[2,2],sebetaXY=a[2,3],p=a[2,3],
           betaXYLCI=c[2,1],betaXYUCI=c[2,2])
}
library(MendelianRandomization)
## IVW method in MendelianRandomization
ivwMR <- function(datMRInput) {
  a <- attributes(MendelianRandomization::mr_ivw(datMRInput))
  b <- attributes(MendelianRandomization::mr_median(datMRInput))
  rbind(data.frame(method="IVW",
              betaXY=a$Estimate,sebetaXY=a$StdError,
              p=a$Pvalue,betaXYLCI=a$CILower,betaXYUCI=a$CIUpper),
       data.frame(method="Weighted median",
              betaXY=b$Estimate,sebetaXY=b$StdError,
              p=b$Pvalue,betaXYLCI=b$CILower,betaXYUCI=b$CIUpper))
}
library(mr.raps)
## MR-RAPS
mrrapsMR <- function(bx,bxse,by,byse){
  a <- mr.raps(b_exp=bx,se_exp=bxse,b_out=by,se_out=byse)
  data.frame(method="MR-RAPS",
           betaXY=a$beta.hat,sebetaXY=a$beta.se,
           p=a$beta.p.value,
           betaXYLCI=a$beta.hat-1.96*a$beta.se,
           betaXYUCI=a$beta.hat+1.96*a$beta.se)
}
library(ggplot2)
library(ggsci)
library(scales)
mypal <- pal_lancet("lanonc",alpha=0.9)(8); mypal

J <- 10; numG <- J
r2GX <- 0.02; FStat <- 20
## Pierce an Burgess. Am J Epidemiol 2013; 178: 1177-84.
```

```
## Calculate the standard deviation of mubetaGX based on explained variance
##   of genetic variants. : sqrt(r2GX/(1-r2GX)
mubetaGX <- 0.0; sebetaGX <- sqrt(r2GX/(1-r2GX))
mubetaGY <- 0.0; sebetaGY <- 0.05
mubetaUX <- 0.0; mubetaUY <- 0.5; pU <- 0.5

mubetaXY <- 2
mubetaGS <- log(runif(numG,min=1.0,max=1.5));  # HR = [1.5,2.5]
mubetaXS <- log(1.50); # HR=1.50
mubetaCS <- log(1.50); # HR=1.50
mubetaYS <- log(2.50); # HR=2.50
mubetaUS <- log(2.00); # HR=1.50

nX <- FStat*(1-r2GX)/r2GX*numG + 1 + numG; nX
nX <- 1e05


SimConExp <- function(mubetaXY,
                      mubetaGS,mubetaXS,mubetaCS,mubetaYS,mubetaUS,
                      ageMinExp=40,ageMaxExp=69,ageMinOut=40,ageMaxOut=89) {
  set.seed(666666)
  ## Original data
  ## SNP-exposure associations
  mafG <- runif(numG,min=0.1,max=0.5)
  G <- do.call("cbind",
               lapply(1:numG, function(j) {
                 (rbinom(nX, 2, mafG[j])-2*mafG[j])/sqrt(2*mafG[j]*(1-mafG[j]))
               }))
  C <- rnorm(nX); U <- rbinom(nX,1,pU)
  # left-truncated normal distribution with risk increasing allele effect
  betaGX <- rtnorm(numG,mu=mubetaGX,sd=sebetaGX,a=0.2);betaGX
  epiX <- rnorm(nX,mean=0,sd=1)
  X <- as.numeric(G %*% matrix(betaGX,ncol=1) + mubetaUX*U + epiX)
  lmFitGX <- function(i) { betaGXhat <- coef(summary(lm(X~G[,i])))[2,1:2] }
  datExp <- do.call("rbind", lapply(1:numG, function(j) lmFitGX(j)) )
  colnames(datExp) <- c("betaGX", "sebetaGX"); cbind(betaGXT=betaGX,datExp)
  ## SNP-outcome associations
  betaGY <- rnorm(numG,mean=mubetaGY,sd=sebetaGY);betaGY
  epiY <- rnorm(nX,mean=0,sd=1)
  Y <- as.numeric(mubetaXY*X + G %*% matrix(betaGY,ncol=1) + mubetaUY*U + epiY)
  lmFitGY <- function(i) { betaGYhat <- coef(summary(lm(Y~G[,i])))[2,1:2] }
  datOut <- do.call("rbind", lapply(1:numG, function(j) lmFitGY(j)) )
  colnames(datOut) <- c("betaGY", "sebetaGY"); cbind(betaGYT=betaGY,datOut)

  ## -- Selected data
  ## Generating survival time
  #generate survival time based on Gompertz model
```

```
gomp.a<-0.0000459053
gomp.b<-0.0876978320
s<- runif(nX)
RR <- exp(mubetaXS*X +
          mubetaCS*C +
          mubetaUS*U +
          mubetaYS*Y +
          as.numeric(G %*% matrix(mubetaGS,ncol=1))); RR
survtime <- log(1-(gomp.b*log(s)/(gomp.a*RR)))/gomp.b; #hist(survtime)

sIdxExp <- survtime>ageMinExp & survtime<ageMaxExp ## Select for exposure GWAS
GSelExp<-G[sIdxExp,];XSelExp<-X[sIdxExp];
YSelExp<-Y[sIdxExp];USelExp<-U[sIdxExp]
lmFitGXSel <- function(i) {
  betaGXhat <- coef(summary(lm(XSelExp~GSelExp[,i])))[2,1:2]
}
datExpSel <- do.call("rbind", lapply(1:numG, function(j) lmFitGXSel(j)) )
colnames(datExpSel) <- c("betaGX", "sebetaGX");
cbind(betaGXT=betaGX,datExpSel)

sIdxOut <- survtime>ageMinOut & survtime<ageMaxOut ## Select for outcome GWAS
GSelOut<-G[sIdxOut,];XSelOut<-X[sIdxOut];
YSelOut<-Y[sIdxOut];USelOut<-U[sIdxOut]
lmFitGYSel <- function(i) {
  betaGYhat <- coef(summary(lm(YSelOut~GSelOut[,i])))[2,1:2]
}
datOutSel <- do.call("rbind", lapply(1:numG, function(j) lmFitGYSel(j)) )
colnames(datOutSel) <- c("betaGY", "sebetaGY");
cbind(betaGYT=betaGY,datOutSel)

## MR analysis
## Full dataset
# res1 <- ivRes(out=Y,exp=X,ins=G,cov=U);res1$idx <- "1SMR: Full"; res1
# res2 <- ivRes(out=YSel,exp=XSel,ins=GSel,cov=USel);
# res2$idx <- paste("1SMR: AgeExp=[",ageMinExp,",",ageMaxExp,"]",
#                  "AgeOut=[",ageMinOut,",",ageMaxOut,"]",sep=""); res2
res3 <- ivwMR(mr_input(bx=datExp[,"betaGX"],bxse=datExp[,"sebetaGX"],
                  by=datOut[,"betaGY"],byse=datOut[,"sebetaGY"]));
res3$idx <- "2SMR: Full"; res3
res4 <- ivwMR(mr_input(bx=datExpSel[,"betaGX"],bxse=datExpSel[,"sebetaGX"],
                  by=datOutSel[,"betaGY"],byse=datOutSel[,"sebetaGY"]))
res4$idx <- paste("2SMR: AgeExp=[",ageMinExp,",",ageMaxExp,"]",
               "AgeOut=[",ageMinOut,",",ageMaxOut,"]",sep=""); res4
res5 <- mrrapsMR(bx=datExp[,"betaGX"],bxse=datExp[,"sebetaGX"],
              by=datOut[,"betaGY"],byse=datOut[,"sebetaGY"])
res5$idx <- "2SMR: Full"; res5
res6 <- mrrapsMR(bx=datExpSel[,"betaGX"],bxse=datExpSel[,"sebetaGX"],
```

```
               by=datOutSel[,"betaGY"],byse=datOutSel[,"sebetaGY"])
  res6$idx <- paste("2SMR: AgeExp=[",ageMinExp,",",ageMaxExp,"]",
                "AgeOut=[",ageMinOut,",",ageMaxOut,"]",sep=""); res6
  # rbind(res1,res2,res3,res4,res5,res6)
  res <- rbind(res3,res4,res5,res6)
  res$PrExp=mean(sIdxExp)
  res$PrOut=mean(sIdxOut)
  res
}


## Scenario 2-2: Focus on mubetaGS and mubetaXY
mubetaXY22 <- c(0,1)
mubetaGS22 <- matrix(log(rep(seq(0.5,2.5,0.1),numG)),ncol=numG);  # HR = [1.5,2.5]
mubetaXS22 <- log(1.0)
mubetaCS22 <- log(1.0)
mubetaYS22 <- log(2.0)
mubetaUS22 <- log(1.0)

resS22 <- data.frame()
for (i in 1:length(mubetaXY22)) {
  resScenario22 <- do.call("rbind",
                      lapply(1:nrow(mubetaGS22),
                          function(j) {
                            a <- SimConExp(mubetaXY=mubetaXY22[i],
                                     mubetaGS=as.numeric(mubetaGS22[j,]),
                                     mubetaXS=mubetaXS22,
                                     mubetaCS=mubetaCS22,
                                     mubetaYS=mubetaYS22,
                                     mubetaUS=mubetaUS22)
                            a$mubetaXY <- mubetaXY22[i]
                            a$mubetaGS <- exp(mubetaGS22[j,1])
                            a$mubetaXS <- exp(mubetaXS22)
                            a$mubetaCS <- exp(mubetaCS22)
                            a$mubetaYS <- exp(mubetaYS22)
                            a$mubetaUS <- exp(mubetaUS22)
                            a
                          }))
  resS22 <- rbind(resS22, resScenario22)
}
resS22



## Scenario 2-3: Focus on mubetaGS and mubetaXY

setDT(resS22);resS22[,`:=`(SBBias=betaXY-mubetaXY)]
```

```
resS12 <- rbindlist(list(resS22),fill=TRUE)
resS12New<-resS12[
  ,`:=`(group=ifelse(mubetaXY==0&mubetaXS==1.0&mubetaCS==1.0&mubetaYS==2.0&mub
etaUS==1.0,"Scenario (a)",
                "Scenario (b)"))]

resS12NewNew <- resS12New[idx != "2SMR: Full"&method == "IVW"]
pS12 <- ggplot(data=resS12NewNew,
            aes(x=mubetaGS,y=SBBias,shape=factor(group),color=factor(group))) +
  theme_classic() +
  theme(legend.title=element_blank(),legend.position=c(0.2,0.2)) +
  geom_point(position=position_dodge(width=0.1),size=2) +
  geom_line(position=position_dodge(width=0.1)) +
  # geom_pointrange(aes(ymin=SBBiasLCI,ymax=SBBiasUCI),
  #               position=position_dodge(width=0.1),size=0.5) +
  geom_hline(aes(yintercept=0)) +
  scale_x_continuous(name=expression(paste("Effect of genetic instruments on survival ",
HR[GS], sep="")),
                breaks=seq(0.5,2.5,by=0.25),
                limits=c(0.45,2.55),expand=c(0,0)) +
  scale_y_continuous(name=expression(paste("Impacts of selection bias on MR estimates ",
hat(beta)[E1D]," - ", beta[E1D],sep="")),
                breaks=seq(-1.2,0.6,0.3),limits=c(-1.2,0.6),expand=c(0,0)) +
  scale_color_lancet() +
  scale_fill_lancet()
pS12


## Scenario 8-1: Focus on mubetaYS and mubetaXY
mubetaXY81 <- c(0,1)
mubetaGS81 <- rep(log(1.25),numG);  # HR = [1.5,2.5]
mubetaXS81 <- log(1.0)
mubetaCS81 <- log(1.0)
mubetaYS81 <- log(seq(0.5,2.5,0.1))
mubetaUS81 <- log(1.0)

resS81 <- data.frame()
for (i in 1:length(mubetaXY81)) {
  resScenario81 <- do.call("rbind",
                    lapply(1:length(mubetaYS81),
                         function(j) {
                           a <- SimConExp(mubetaXY=mubetaXY81[i],
                                    mubetaGS=mubetaGS81,
                                    mubetaXS=mubetaXS81,
                                    mubetaCS=mubetaCS81,
                                    mubetaYS=mubetaYS81[j],
```

```
                                   mubetaUS=mubetaUS81)
                            a$mubetaXY <- mubetaXY81[i]
                            a$mubetaGS <- exp(mubetaGS81[1])
                            a$mubetaXS <- exp(mubetaXS81)
                            a$mubetaCS <- exp(mubetaCS81)
                            a$mubetaYS <- exp(mubetaYS81[j])
                            a$mubetaUS <- exp(mubetaUS81)
                            a
                           }))
  resS81 <- rbind(resS81, resScenario81)
}
resS81


## Scenario 8-2: Focus on mubetaYS and mubetaXY
mubetaXY82 <- c(0,1)
mubetaGS82 <- rep(log(1.0),numG);  # HR = [1.5,2.5]
mubetaXS82 <- log(1.5)
mubetaCS82 <- log(1.0)
mubetaYS82 <- log(seq(0.5,2.5,0.1))
mubetaUS82 <- log(1.0)

resS82 <- data.frame()
for (i in 1:length(mubetaXY81)) {
  resScenario82 <- do.call("rbind",
                        lapply(1:length(mubetaYS82),
                            function(j) {
                             a <- SimConExp(mubetaXY=mubetaXY82[i],
                                    mubetaGS=mubetaGS82,
                                    mubetaXS=mubetaXS82,
                                    mubetaCS=mubetaCS82,
                                    mubetaYS=mubetaYS82[j],
                                    mubetaUS=mubetaUS82)
                             a$mubetaXY <- mubetaXY82[i]
                             a$mubetaGS <- exp(mubetaGS82[1])
                             a$mubetaXS <- exp(mubetaXS82)
                             a$mubetaCS <- exp(mubetaCS82)
                             a$mubetaYS <- exp(mubetaYS82[j])
                             a$mubetaUS <- exp(mubetaUS82)
                             a
                            }))
  resS82 <- rbind(resS82, resScenario82)
}
resS82


setDT(resS81);resS81[,`:=`(SBBias=betaXY-mubetaXY)]
```

```r
setDT(resS82);resS82[,`:=`(SBBias=betaXY-mubetaXY)]

resS78 <- rbindlist(list(resS81,resS82[mubetaXY==1]),fill=TRUE)
resS78New<-resS78[
  ,`:=`(group=ifelse(mubetaXY==0&mubetaXS==1.0&mubetaCS==1.0&mubetaGS==1.25&mu
betaUS==1.0,"Scenario (a)",

ifelse(mubetaXY==1&mubetaXS==1.0&mubetaCS==1.0&mubetaGS==1.25&mubetaUS==1.0,
"Scenario (b)","Scenario (c)")))]

resS78NewNew <- resS78New[idx != "2SMR: Full"&method == "IVW"]
pS78 <- ggplot(data=resS78NewNew,
            aes(x=mubetaYS,y=SBBias,shape=factor(group),color=factor(group))) +
  theme_classic() +
  theme(legend.title=element_blank(),legend.position=c(0.2,0.2)) +
  geom_point(position=position_dodge(width=0.1),size=2) +
  geom_line(position=position_dodge(width=0.1)) +
  # geom_pointrange(aes(ymin=SBBiasLCI,ymax=SBBiasUCI),
  #               position=position_dodge(width=0.1),size=0.5) +
  geom_hline(aes(yintercept=0)) +
  scale_x_continuous(name=expression(paste("Effect of outcome on survival ", HR[DS],
sep="")),
                breaks=seq(0.5,2.5,by=0.25),
                limits=c(0.45,2.55),expand=c(0,0)) +
  scale_y_continuous(name=expression(paste("Impacts of selection bias on MR estimates ",
hat(beta)[E1D]," - ", beta[E1D],sep="")),
                breaks=seq(-1.2,0.6,0.3),limits=c(-1.2,0.6),expand=c(0,0)) +
  scale_color_lancet() +
  scale_fill_lancet()
pS78



savePDF <- function(name,figure) {
  pdf(file=paste(getwd(),"/Result/",name,sep=""),width=7,height=5,onefile=FALSE)
  print(figure)
  dev.off()
}
pS12;savePDF(name="SBG-Updated-20211019.pdf",figure=pS12)
pS78;savePDF(name="SBY-Updated-20211019.pdf",figure=pS78)
```
