# Supplementary material for: Credible Mendelian Randomization Studies in the Presence of Selection Bias Using Control Exposures
Source: Front Genet. 2021 Nov 24;12:729326. doi: 10.3389/fgene.2021.729326 (PMC8652250; doi:10.3389/fgene.2021.729326)
Supplement: Supplementary file 2 [file DataSheet1.PDF]

Supplementary material of

"Credible Mendelian randomization studies in the presence of selection bias using control exposure"

Zhao Yang, C Mary Schooling, Man Ki Kwok

Contents

Data generation process 2

Simulation study 3

Simulation results 6

References 9

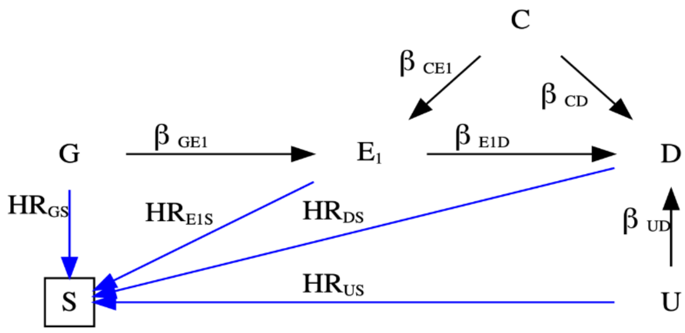

Supplementary Figure S1. Directed acyclic graph (DAG) illustrating possible data-generating processes underlying selection bias in Mendelian randomization (MR). G: the genetic instruments; E_1: the exposure of interest; C: the confounder of the E_1-D association; D: the outcome of interest; U: the unmeasured confounder that affects both D and the survival S; β_{GE1}: the genetic associations on E_1; β_{E1D}: the causal effect of E_1 on D that is the estimate of interest; β_{UD}: the effect of U on D; HR_{GS}: the relative hazard of G on the survival S of the underlying population; HR_{E1S}: the relative hazard of E_1 on the survival S; HR_{DS}: the relative hazard of D on the survival S; HR_{US}: the relative hazard of U on the survival S. Moreover, HR_{CS}: the relative hazard of C on the survival S, which is not showed in the DAG.

Data generation process

We are interested in estimating the causal effect (i.e., β_{E1D}) of E_1 on D in selected samples influenced by selecting on the genetic instruments G_{E1}or exposure E_1 and outcome D or the unmeasured confounder U causing the outcome and the competing risks CR, as shown in **Figures 1(c)-(d)**. As such, selecting survivors may violate the IV3 assumption and induce selection bias. For simplicity, we modelled the impact of U on survival directly instead of explicitly considering the competing risks CRs. We induced selection bias by selecting study participants among survivors of the original birth cohorts who formed the population until study recruitment, as shown in **Figure S1**. We assumed that the survival of the underlying population was influenced by G (i.e., $HR_{GS} = \exp(\beta_{GS})$), E_1 (i.e., $HR_{E1S} = \exp(\beta_{E1S})$), C (i.e., $HR_{CS} = \exp(\beta_{CS})$), D (i.e., $HR_{DS} = \exp(\beta_{DS})$), and U (i.e., $HR_{US} = \exp(\beta_{US})$), reflecting by hazard ratio in per-unit change. Thus, selection bias arising from the sample selection among survival till study recruitment will be governed by HR_{GS}, HR_{E1S}, HR_{CS}, HR_{DS}, and HR_{US}. The impact of selection bias on MR estimates from sample

selection conditioning on genetic variants and the outcome D can be achieved by setting $HR_{GS} \neq 1, HR_{E1S} = 1, HR_{CS} = 1, HR_{DS} \neq 1$, and $HR_{US} = 1$.

We generated survival time (T) for the underlying population via a Gompertz model presented in Smit et al.'s paper (1). Specifically, the Gompertz survival model was derived from the 2016 mortality data of the United States based on the Human Mortality Database within an R-package of *MortalityLaws*. Based on the generated survival time, we induced selection bias by allocating people aged 40 to 69 years to the exposure GWAS and those aged 40 to 89 years to the outcome GWAS among survivors, constructing the two-sample MR setting. The reason here is that study participants in the exposure GWAS were always younger than in the outcome GWAS; i.e., risk factors (exposure) causes diseases (outcome).(2) However, this is not necessary for selection bias to occur, but is used as a simplification here. Often MR concerns continuous exposures and all-or-nothing outcomes, continuous variables are usually less biased by selection bias than all-or-nothing variables, so an age difference between the population generating the exposure and outcome is not required for selection bias to occur.

Simulation study

To illustrate selection bias in MR, as shown in **Figures 1(c)-(d)**, we performed extensive simulation studies concerning IVW because it was the most widely used method.(3)

For the ith subject, we simulated data on jth (i.e., $j = 1, \ldots, J$) genetic variant G_{ij} (which is coded 0, 1, and 2 to indicate the number of copies of relevant risk allele), exposure E_{1i}, and outcome D_i in the presence of an unobserved exposure-outcome confounder C_i. We modelled the independent genetic variants via a Binominal distribution (e.g., $G_{ij} \sim \mathrm{Bin}(maf_j)$) with the minor allele frequency drawn from a Uniform distribution (e.g., $maf_j \sim \mathrm{Unif}(0.1, 0.5)$).

In general, there are a number of unobserved exposure-outcome confounders (i.e., C_i in **Figure 1**). We supposed C is a continuous variable with mean and variance being 0 and 1, respectively. In the presence of selection bias, we assumed that no unmeasured exposure-outcome confounders (i.e., C_i) exist (i.e., $\beta_{CE1} = \beta_{CD} = 0$), but a single binary confounder (U_i) that affects both the outcome D_i and the competing risk (i.e., CR_i), as shown in **Figure**

1(c). For simplicity, we modelled the relative hazard of U on survival (i.e., HR_{US}) directly rather than via CRs, with the relative hazard of C on survival modelled as HR_{CS}. Specifically, we simulated U via a binominal distribution with a rate of 0.5. That is, the prevalence of U among the underlying population was 0.5. The effect of genetic variant G_j on unobserved confounder U is represented by β_{jGU}, in which non-zero of β_{jGU} indicates an invalid IV of G_j. Herein, we assumed that all instruments were valid IVs; that is, $\beta_{jGU} = 0$ for $j = 1, \ldots, J$.

The exposure E_{1i} is linear in the genetic variants, unobserved confounder U_i, and an independent error term ϵ_{iE1}, in which the genetic exposure association is represented by β_{jGE1}, with the confounding effect of U_i represented by β_{UE1}. We simulated the genetic-exposure association β_{jGE1} using a left-sided truncated normal distribution at 0.2 described by Slob and Burgess (4) to ensure risk increasing allele effects after standardization. The variance of the distribution is chosen to make the explained variation of exposure by all genetic variants be around 5%.(5)

The outcome D_i is a linear function of additive direct effect of G_{ij}, E_{1i}, U_i, and an independent error term ϵ_{iD}, in which the confounding effect of U_i represented by β_{UD}. Thus, the direct effect of genetic variant G_j ($j = 1, \ldots, J$) on D is represented by β_{jGD}, in which non-zero of β_{jGD} indicates the violation of IV3 assumption. For simplify, we set $\beta_{UE1} = 0, \beta_{UD} = 0.5$, and $\beta_{jGD} \sim N(0, 0.05^2)$ for $j = 1, \ldots, J$. Thus, the causal effect of exposure E_1 on outcome D is represented by β_{E1D}. In addition, the ϵ_{iE} and ϵ_{iD} are random errors and simulated through a standardized normal distribution, respectively. As such, the data generation modelling can be written mathematically, as follows.

$$G_{ij} \sim \text{Bin}\left(maf_j\right) \text{ independently, with } maf_j \sim \text{Unif}(0.1, 0.5),$$

$$C_i \sim N(0,1);\ U_i \sim \text{Bin}(0.5),$$

$$E_{1ij} = \sum_{j=1}^{J} \beta_{jGE1} G_{ij} + \beta_{CE1} C_i + \beta_{UE1} U_i + \epsilon_{iE1},$$

$$D_i = \sum_{j=1}^{J} \beta_{jGD} G_{ij} + \beta_{DE1} E_{1i} + \beta_{CD} D_i + \beta_{UD} U_i + \epsilon_{iD},$$

$$HR_i = \exp\left(\sum_{j=1}^{J} \beta_{jGS} G_{ij} + \beta_{E1S} E_i + \beta_{DS} D_i + \beta_{CS} C_i + \beta_{US} U_i\right),$$

$$T_i = \frac{\log\left(1 - \frac{\gamma \log(S_i)}{\lambda * HR_i}\right)}{\gamma}, \lambda = 0.0000459053, \gamma = 0.0876978320, S_i \sim \text{Unif}(0,1),$$

$T_i \in [40,69]$ for the exposure GWAS, $T_i \in [40,89]$ for the outcome GWAS,

$\epsilon_{iE1}, \epsilon_{iD} \sim N(0,1)$ independently.

We emphasized scenarios as depicted in Figure 1 without violation of any required assumptions in MR, given NULL ($\beta_{E1D} = 0$) or positive ($\beta_{E1D} = 1$) association of E_1 with D. That is,

(1) Survival of the underlying population were mainly influenced by G_j ($j = 1, \dots, J$), with a fixed effect of D but for C, E_1 and U on survival; i.e., $HR_{E1S} = 1.0$, $HR_{CS} = 1.0$, $HR_{DS} = 2.0$, $HR_{US} = 1.0$;

(2) Survival of the underlying population were mainly influenced by D, with a fixed effect of E_1 but for G_j ($j = 1, \dots, J$), C, and U on survival; i.e., $HR_{GjS} = 1.25$, $HR_{E1S} = 1.0$, $HR_{CS} = 1.0$, $HR_{US} = 1.0$;

(3) Survival of the underlying population were mainly influenced by D, with a fixed effect of E_1 but for G_j ($j = 1, \dots, J$), U, C, and U on survival; i.e., $HR_{GjS} = 1.0$, $HR_{E1S} = 1.5$, $HR_{CS} = 1.0$, $HR_{US} = 1.0$.

We simulated data on $J = 10$ genetic variants. Summary genetic associations were calculated for exposure and outcome separately based on the selected samples, referred to as the two-sample MR.(5) We set the total sample size to be 100,000 to ensure adequate power. Notably, due to the different time lags between generic randomization at conception and the study recruitment for the exposure (i.e., 40-69 years) and outcome (i.e., 40-89 years) GWAS, sample sizes for genetic-exposure and genetic-outcome associations may vary, depending on the actual situations. All simulations were conducted in R (version 3.6.3).

Simulation results

Figure 3 and **Supplementary Figure S1** show the impact of selection bias arising from selecting samples conditioning on genetic instruments G and outcome D, with no effects of either exposure E_1 or the shared confounder U of D mediated by competing risks on survival of the underlying population (i.e., birth cohort) based on simulation studies. As expected, selecting samples conditioning on genetic variant G and outcome D of interest induces selection bias, with its impacts varying depending on the relative hazard of G and D on survival of the underlying population. Given summary statistics obtained from the original exposure and outcome GWASs, it seems not easy to recover the true causal estimate from the observed MR estimates in two-sample MR settings due to the essence missing people before the recruitment of the original GWASs. However, our proposal provides a valuable approach to assessing credible MR estimates in the presence of selection bias from selection of survivors.

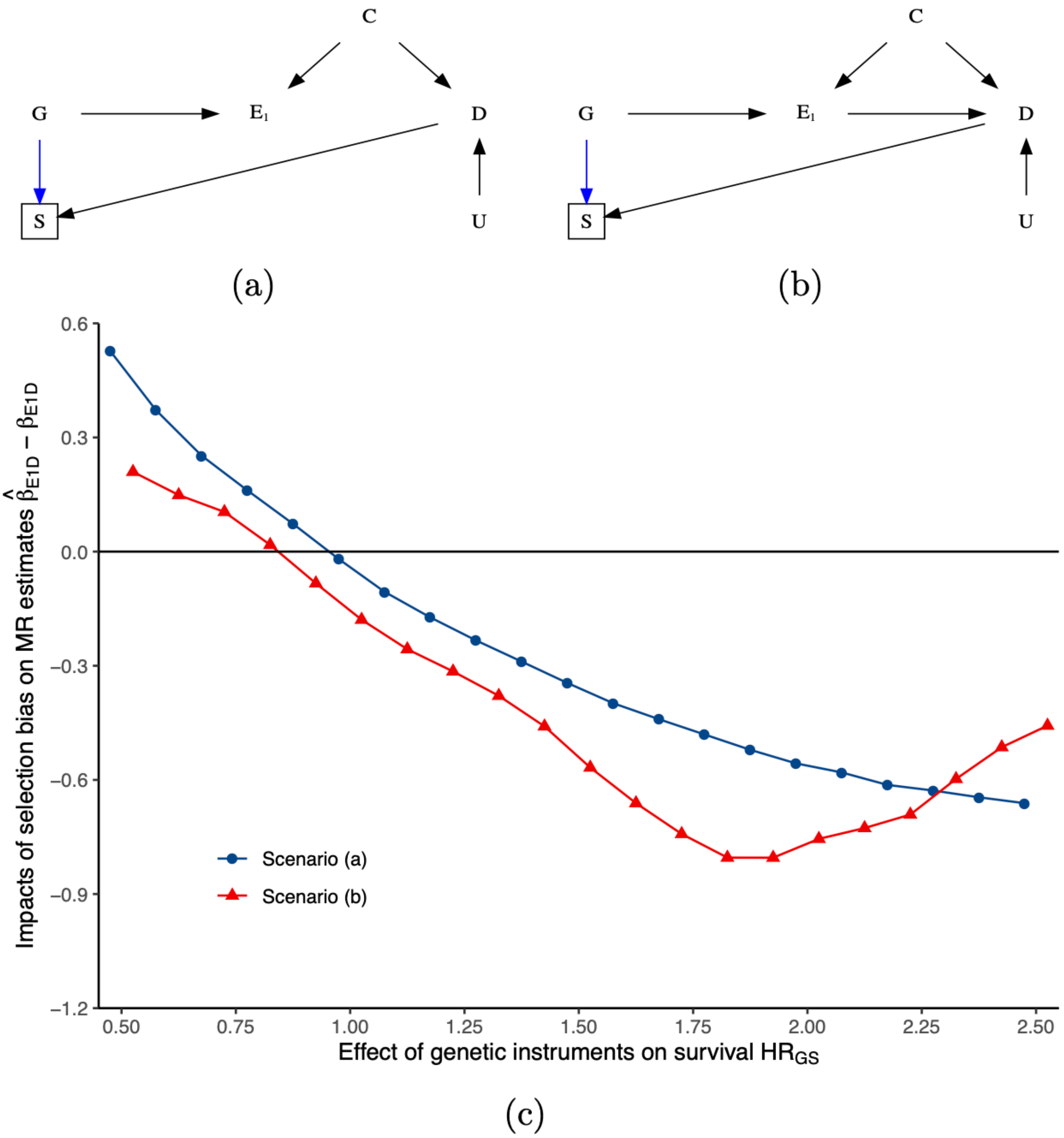

Figure 3. The impacts of selection bias (i.e., $\hat{\beta}_{E1D} - \beta_{E1D}$) on two-sample Mendelian randomization (MR) estimates of the exposure E_1-outcome D association using the inverse variance weighted method in terms of various relative hazard (HR) of per-unit change in genetic variant G (i.e., HR_{GS}) with fixed effects of either D (i.e., HR_{DS}) on survival of underlying population based on simulation studies, with more details presented in **Supplementary Material 1**. The upper panel (a)-(b) show scenarios that may happen in practice. The lower panel (c) shows the impacts of selection bias on MR estimates under each scenario. R codes for reproducing these results can be found in **Supplementary Material 2**.

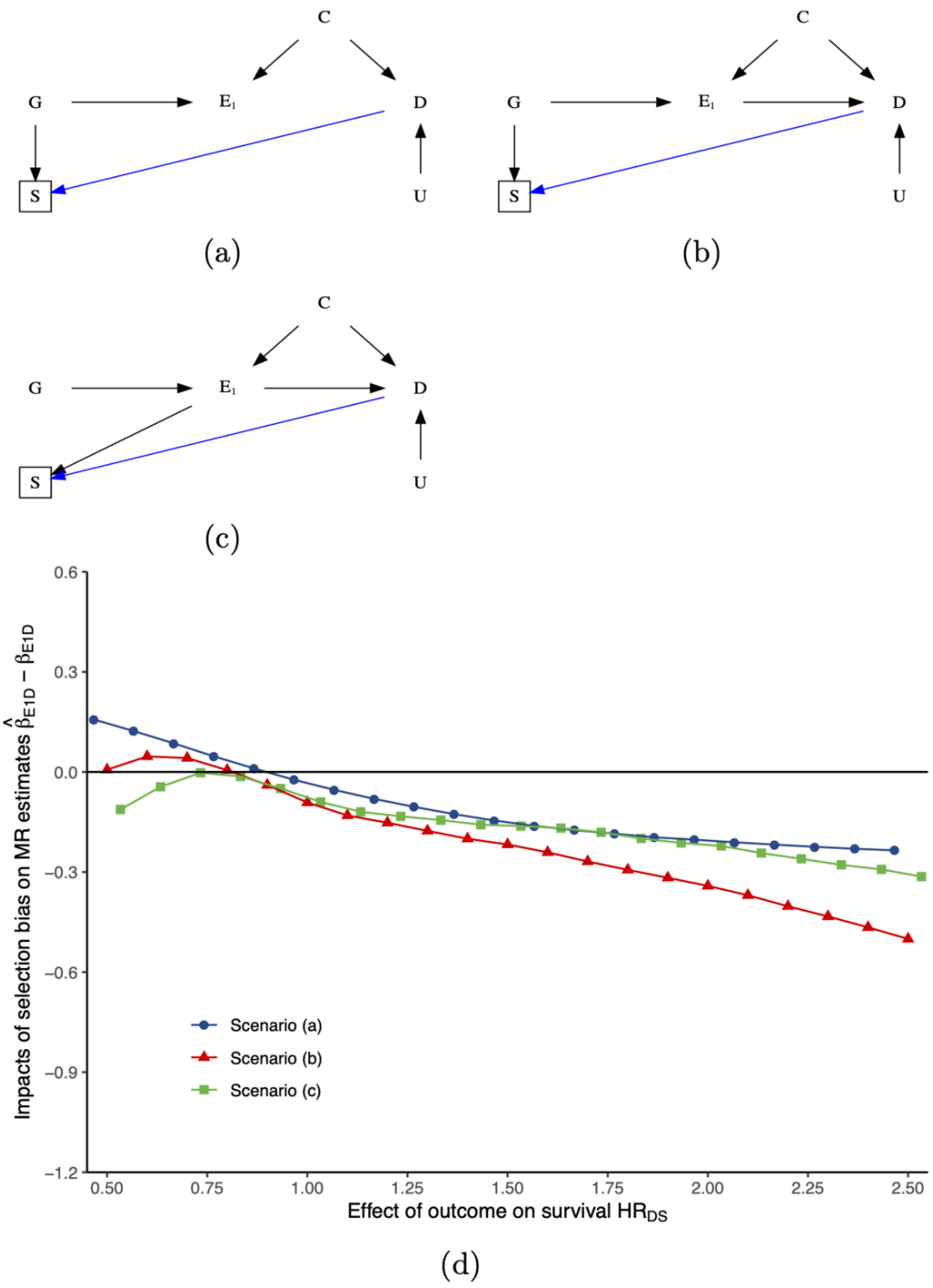

Supplementary Figure S1. The impacts of selection bias (i.e., $\hat{\beta}_{E1D} - \beta_{E1D}$) on two-sample Mendelian randomization (MR) estimates of the exposure E_1-outcome D association using the inverse variance weighted method in terms of various relative hazard (HR) of per-unit change in D with fixed effects of either genetic instruments G (i.e., HR_{GS}) or E_1 (i.e., HR_{E1S}) on survival of underlying population based on simulation studies. The upper panel (a)-(c) show six scenarios that may happen in practice. The lower panel (d) shows the impacts of selection bias on MR estimates under each scenario.

References

1. Smit RAJ, Trompet S, Dekkers OM, Jukema JW, le Cessie S. Survival bias in Mendelian randomization studies: A threat to causal inference. *Epidemiology* (2019) 30(6):813-6. Epub 2019/08/03. doi: 10.1097/EDE.0000000000001072. PubMed PMID: 31373921; PubMed Central PMCID: PMCPMC6784762.

2. Schooling CM, Lopez PM, Yang Z, Zhao JV, Au Yeung SL, Huang JV. Use of multivariable Mendelian randomization to address biases due to competing risk before recruitment. *Front Genet* (2020) 11:610852. Epub 2021/02/02. doi: 10.3389/fgene.2020.610852. PubMed PMID: 33519914; PubMed Central PMCID: PMCPMC7845663.

3. Hemani G, Zheng J, Elsworth B, Wade KH, Haberland V, Baird D, et al. The MR-Base platform supports systematic causal inference across the human phenome. *Elife* (2018) 7. Epub 2018/05/31. doi: 10.7554/eLife.34408. PubMed PMID: 29846171; PubMed Central PMCID: PMCPMC5976434.

4. Slob EAW, Burgess S. A comparison of robust Mendelian randomization methods using summary data. *Genet Epidemiol* (2020) 44(4):313-29. Epub 2020/04/07. doi: 10.1002/gepi.22295. PubMed PMID: 32249995; PubMed Central PMCID: PMCPMC7317850.

5. Pierce BL, Burgess S. Efficient design for Mendelian randomization studies: subsample and 2-sample instrumental variable estimators. *Am J Epidemiol* (2013) 178(7):1177-84. Epub 2013/07/19. doi: 10.1093/aje/kwt084. PubMed PMID: 23863760; PubMed Central PMCID: PMCPMC3783091.
